# Advances and challenges in novel drug delivery systems for glioma therapy

**DOI:** 10.3389/fphar.2025.1655241

**Published:** 2025-08-21

**Authors:** Peipei Ma, Yongkang Li, Yingrui Gu, Hui Zeng, Hongwei Xiang, Zhenzhen Cao, Yankun Han, Yong Cui, Haixiao Liu

**Affiliations:** ^1^ Department of Neurosurgery, Tangdu Hospital, The Fourth Military Medical University, Xi’an, Shaanxi, China; ^2^ Basic Medical College of The Fourth Military Medical University, Xi’an, Shaanxi, China; ^3^ Department of Neurosurgery, Seventh People’s Hospital of Shanghai University of Traditional Chinese Medicine, Shanghai, China; ^4^ Department of Neurosurgery, Shanghai Fourth People’s Hospital, Tongji University, Shanghai, China; ^5^ Department of Biomedical Engineering, Fourth Military Medical University, Xi’an, Shaanxi, China

**Keywords:** glioma, drug delivery systems, blood-brain barrier, drug conjugates, focused ultrasound

## Abstract

Glioma therapy faces substantial challenges primarily due to the restrictive nature of the blood-brain barrier (BBB), limiting effective drug penetration and reducing therapeutic efficacy. Recent advancements in novel drug delivery systems (DDS), including exosome-mediated carriers, drug conjugates, and ultrasound-assisted delivery, have demonstrated promising results in overcoming these limitations. Exosomes offer superior biocompatibility, efficient BBB crossing, and natural cellular targeting capabilities; drug conjugates enable highly selective drug delivery through tumor-specific ligands; and ultrasound-assisted systems transiently disrupt the BBB to permit greater drug entry. Despite encouraging preclinical and early clinical outcomes, significant translational barriers remain. Challenges such as exosome manufacturing scalability, conjugate stability, and immunogenicity, as well as the optimization of ultrasound protocols, must be thoroughly addressed to achieve clinical translation. Overcoming these hurdles requires ongoing multidisciplinary collaboration and rigorous clinical evaluation. Continued progress in refining these innovative DDS approaches holds the potential to markedly improve therapeutic outcomes and patient prognosis in glioma treatment.

## 1 Introduction

**FIGURE 1:**
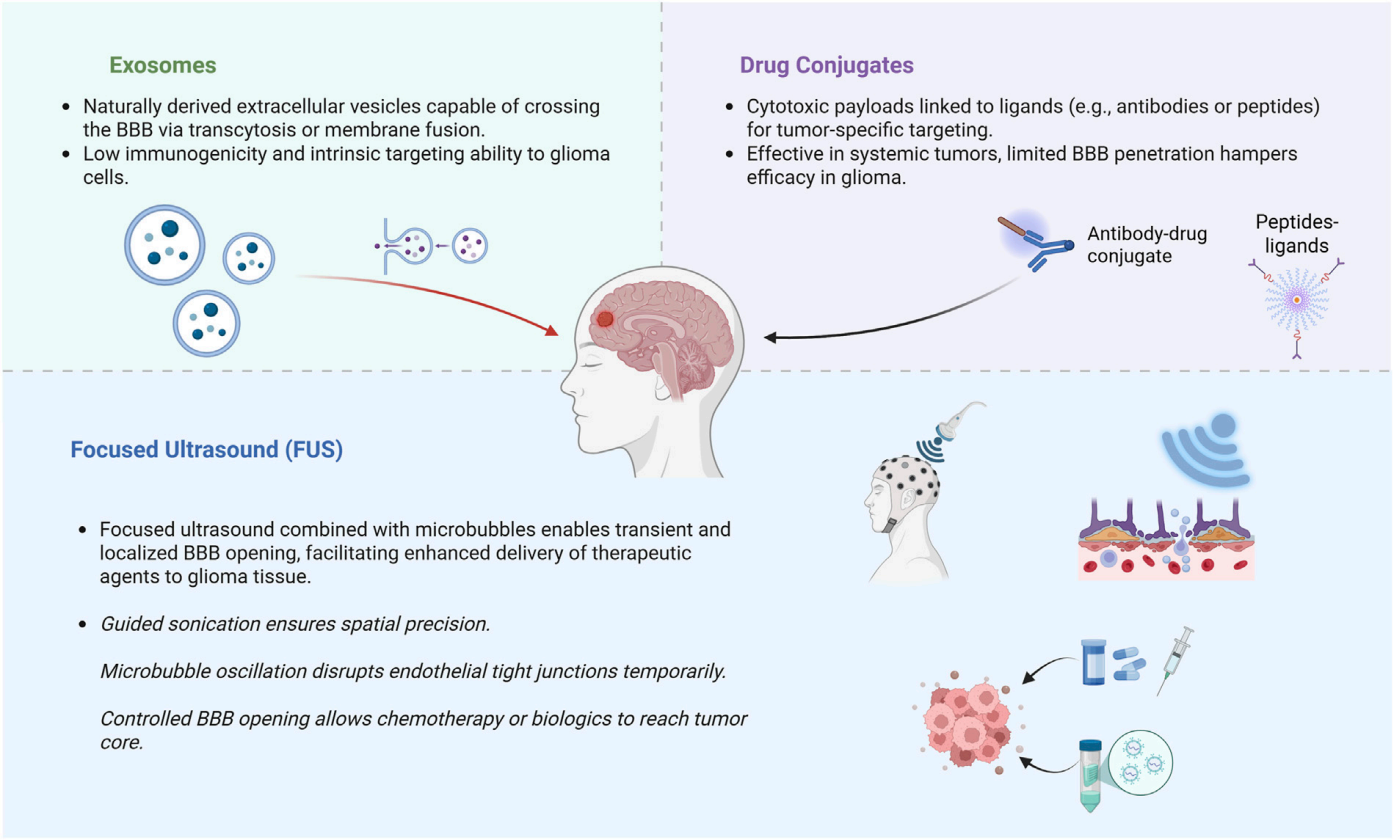
Schematic representation of three major emerging strategies to bypass or transiently open the blood-brain barrier (BBB) in glioma therapy. Exosome-mediated delivery exploits endogenous vesicle transport mechanisms. Drug conjugates enable tumor-specific targeting. Focused ultrasound (FUS) transiently disrupts BBB integrity using microbubble-enhanced sonication. Created in https://BioRender.com.

Gliomas are diffuse and aggressive primary brain neoplasms originating from glial cells. Glioblastoma (GBM), the predominant and most aggressive glioma, constitutes approximately fifty percent of all malignant brain tumors and remains essentially incurable (Ostrom et al., 2022). Despite extensive surgical resection followed by temozolomide chemoradiotherapy, patient prognoses indicate a median survival of merely 15 months, with five-year survival rates falling below 10% (Stupp et al., 2005; Ostrom et al., 2022). This poor prognosis reflects both the infiltrative nature of GBM and the failure of systemic therapies to adequately reach residual tumor cells. In particular, the blood-brain barrier (BBB) is a major obstacle that prevents most drugs from penetrating into the brain. Over 98% of small-molecule drugs and essentially all larger biologics cannot cross the intact BBB, so many promising chemotherapeutics never achieve therapeutic concentrations in the tumor (Lai et al., 2024). Even the standard alkylating agent temozolomide is limited to 20% of its systemic levels in the brain due to efflux transporters at the BBB (ter Linden et al., 2024). Consequently, there is a need for novel drug delivery strategies to overcome the BBB and improve the treatment of glioma. A variety of innovative delivery systems are under active investigation to circumvent these barriers. Exosomes, naturally occurring nanoscale extracellular vesicles, have emerged as biocompatible carriers capable of traversing the BBB and delivering payloads, which specifically refers to therapeutic agents such as chemotherapeutic drugs or cytotoxins, to brain tumors (Khatami et al., 2023; Rana et al., 2025). Another strategy involves drug conjugates, which denote constructs wherein therapeutic agents are chemically linked to targeting ligands or antibodies to achieve selective drug delivery to tumor tissues, such as antibody–drug conjugates, which use tumor-targeting antibodies or ligands to selectively deliver potent cytotoxic compounds to glioma cells. Early clinical studies with antibody–drug conjugates against GBM-associated antigens have shown some promise, though BBB penetration remains a challenge (Parakh et al., 2021). Finally, ultrasound-assisted delivery techniques offer a noninvasive means to transiently open the BBB. Focused ultrasound (FUS) combined with microbubbles can create reversible BBB disruptions, allowing intravenously administered drugs or even gene therapies to permeate into the tumor bed at higher concentrations (Zhu et al., 2024). Preliminary clinical trials employing FUS in patients with high-grade gliomas have established the safety and viability of this method to improve medicine delivery to brain tumors. Overcoming the BBB and improving drug localization in gliomas are critical goals for advancing therapy. Emerging drug delivery systems, including exosome-based carriers, targeted drug conjugates, and focused ultrasound-facilitated delivery, show significant potential to surmount the clinical challenges of GBM (Figure 1). These innovative approaches aim to maximize therapeutic efficacy in the brain while minimizing systemic toxicity, offering hope for better outcomes in this invariably lethal cancer.

## 2 Exosome-mediated drug delivery for glioma therapy

Exosomes are cell-derived nanoscale extracellular vesicles, typically 30–150 nm in diameter, that naturally carry proteins, lipids, and RNAs between cells. Their endogenous origin endows them with high biocompatibility and minimal immunogenicity, and importantly, they can traverse biological barriers like the BBB (Rana et al., 2025). These characteristics make exosomes highly attractive as brain drug delivery vehicles. In contrast to synthetic nanoparticles, exosomes offer a “natural” delivery platform with a high drug-loading capacity and intrinsic stability, capable of deep tissue penetration. They can be engineered for controlled payload release and show excellent biodegradability with low systemic toxicity (Luo et al., 2023). Taken together, these features position exosomes as promising nanocarriers to ferry therapeutics into the brain tumor milieu. A growing body of preclinical studies demonstrates the utility of exosomes for targeted glioma therapy. Exosomes have been loaded with conventional chemotherapeutics such as doxorubicin and paclitaxel to exploit their ability to cross the BBB and selectively deliver drugs to tumor sites (Tian et al., 2014; Luo et al., 2023; Rana et al., 2025). In one notable example, dendritic cell-derived exosomes were engineered to display an α_v integrin-targeting peptide (iRGD) on their surface, enabling them to home to glioma tissue. These iRGD-functionalized exosomes, loaded with doxorubicin, achieved efficient drug accumulation in tumors and significantly suppressed tumor growth *in vivo* with minimal off-target toxicity (Tian et al., 2014). Such findings highlight that exosomal delivery can enhance chemotherapeutic efficacy against gliomas by increasing drug uptake in cancer cells while reducing systemic side effects. Clinically, the targeted and biocompatible nature of exosomes holds great promise for reducing systemic side effects and enhancing patient quality of life, particularly compared with traditional chemotherapy approaches. Beyond small-molecule drugs, exosomes have also been loaded with curcumin and other bioactive compounds, showing improved anti-tumor activity in brain tumor models (Mousavi et al., 2023). Exosomes are equally compelling as carriers for nucleic acid therapies in glioma. They protect RNA cargo from degradation and can shuttle it across the BBB into tumor cells. For instance, mesenchymal stem cell–derived exosomes loaded with a tumor-suppressive microRNA (miR-124a) were shown to effectively deliver the miRNA into glioma stem cells, causing reduced proliferation and increased apoptosis. In an intracranial glioma mouse model, treatment with miR–124a–bearing exosomes resulted in markedly prolonged survival (Lang et al., 2018). This and similar studies illustrate that exosome-mediated transfer of therapeutic microRNAs or siRNAs can potently inhibit glioma growth and invasion. Investigators have also experimented with exosomal delivery of therapeutic mRNAs and even gene-editing cargo, exploiting the vesicles’ natural targeting capabilities to achieve gene modulation within brain tumors. Moreover, exosome surfaces can be functionalized with targeting ligands against tumor-specific receptors, further enhancing selective uptake by glioma cells and improving delivery precision. Despite these encouraging advances, major translational challenges must be overcome before exosome-based glioma therapies can reach the clinic. A foremost issue is scalability, which produces clinical-grade exosomes in sufficient quantities and at a reasonable cost, remains difficult, given the limited yield from cell cultures and the complex purification required. Exosome preparations are also inherently heterogeneous; their size, composition, and cargo can vary depending on the cell source and isolation methods, complicating standardization. Ensuring batch-to-batch consistency in exosomal drug loading and delivery is therefore challenging. Key hurdles include developing large-scale production and purification protocols, improving cargo loading efficiency, and maintaining product uniformity and stability (Luo et al., 2023). Additional concerns involve quality control, such as removing contaminants like co-isolated proteins or other vesicles, and verifying that engineered exosomes retain their targeting functionality and therapeutic payload through storage and administration. Notably, no exosome-based drug delivery product has yet obtained regulatory approval, reflecting the gap between experimental promise and clinical translation. Ongoing research is addressing these issues by exploring bioreactor-based exosome production, refined isolation techniques, and rigorous characterization methods. In summary, exosome-mediated drug delivery offers a novel and biologically inspired strategy for glioma treatment, but careful engineering and process development are required to surmount manufacturing and reproducibility challenges. With continued innovation, exosomal nanocarriers hold the potential to achieve targeted, effective glioma therapy that capitalizes on their natural advantages while meeting the safety and consistency standards for clinical use (Kim et al., 2024).

## 3 Drug conjugates and targeted therapy for glioma

Drug conjugates are designed to selectively deliver potent therapeutics to tumor cells by coupling a cytotoxic agent to a targeting moiety. Antibody–drug conjugates (ADCs) consist of a monoclonal antibody specific for a tumor-associated cell-surface antigen, linked via a chemical linker to a highly potent cytotoxic drug (Lian et al., 2021). This approach allows targeted delivery of the drug into antigen-expressing cells, ideally sparing normal tissue. Peptide–drug conjugates (PDCs) operate on a similar principle, using a targeting peptide instead of an antibody. PDCs can leverage peptides that bind receptors overexpressed on glioma cells or that mediate BBB transcytosis, thereby shuttling drugs into the brain. For example, a 19-amino-acid peptide, Angiopep-2, has been conjugated to paclitaxel (ANG1005) to target low-density lipoprotein receptor-related protein 1 (LRP1), enabling carrier-mediated transport of paclitaxel across the BBB (Kurzrock et al., 2012). In ligand-based delivery systems, endogenous ligands or other affinity molecules guide therapeutics to tumor cells. Notably, interleukins and growth factors have been used to target their upregulated receptors on gliomas. For instance, IL-13 or IL-4 cytokines fused to bacterial toxins can direct cytotoxic payloads to glioma cells overexpressing IL-13Rα2 or IL-4R (Mintz et al., 2002; Suzuki et al., 2015). Similarly, transferrin has been employed to target transferrin receptors on glioma and endothelial cells to facilitate drug uptake into the tumor compartment (Johnsen et al., 2017). These conjugate strategies thus aim to enhance specificity and brain uptake of anti-glioma drugs. Several glioma-targeting conjugates have shown promising preclinical activity. Epidermal growth factor receptor (EGFR) is amplified or mutated (EGFRvIII variant) in a large subset of glioblastomas, making it a prime target for ADC therapy. An EGFR-directed ADC, depatuxizumab mafodotin (Depatux-M, formerly ABT-414), which uses the mAb806 antibody against a tumor-specific EGFR epitope, demonstrated potent anti-tumor effects in preclinical glioma models with EGFR overexpression, EGFR amplification, or EGFRvIII mutations (Van Den Bent et al., 2020; von Achenbach et al., 2024; Lassman et al., 2023). Early-phase clinical studies of Depatux-M in recurrent EGFR-amplified glioblastoma reported notable disease control rates, especially when combined with temozolomide (Van Den Bent et al., 2020). However, a randomized phase III trial in newly diagnosed EGFR-amplified glioblastoma found no significant overall survival benefit from adding Depatux-M to standard chemoradiotherapy, and further development of the drug was terminated (Lassman et al., 2023). One reason for its tolerability was the use of the mAb806 antibody. This antibody selectively binds a cryptic epitope exposed exclusively on tumor EGFR (when highly overexpressed or in the EGFRvIII variant), sparing normal tissues that express only wild-type EGFR. This tumor-selective binding enabled Depatux-M to avoid the typical skin and gastrointestinal toxicities seen with conventional EGFR inhibitors. Another EGFR-targeted conjugate, AMG 595, was designed specifically against the EGFRvIII mutant. AMG 595 (an anti-EGFRvIII antibody conjugated to a maytansinoid) induced potent anti-glioma activity in EGFRvIII-expressing xenograft models. In a phase I trial for recurrent EGFRvIII-positive glioblastoma, AMG 595 exhibited favorable pharmacokinetics and tolerability, with a few patients achieving partial tumor responses, though most had stable disease as the best outcome (Rosenthal et al., 2019). These studies indicate the potential of EGFR/EGFRvIII-targeted ADCs while also highlighting the need for further optimization. Interleukin-13 receptor alpha 2 (IL-13Rα2) is another compelling target for glioma conjugate therapy. IL-13Rα2 is overexpressed in the majority of glioblastomas but is essentially absent from normal brain tissue (Liang et al., 2022), making it an almost tumor-exclusive marker. Conjugates exploiting this receptor have shown encouraging preclinical results. An anti-IL13Rα2 ADC was found to exert potent cytotoxic effects in IL13Rα2-positive diffuse intrinsic pontine glioma cell models and *in vivo* tumor models, with drug activity correlating strongly with IL13Rα2 expression levels (Lian et al., 2021). Furthermore, recombinant immunotoxins composed of IL-13 ligand fused to *Pseudomonas* exotoxin have demonstrated the ability to selectively kill IL13Rα2-expressing glioma cells. In clinical evaluations, locoregional infusion of IL13-PE toxin conjugate showed signs of antitumor activity in recurrent glioblastoma, though therapy was complicated by dose-limiting neurotoxicity and immunogenic reactions (Heiss et al., 2018). Beyond cytokine receptors, other glioma-associated markers have been explored. For example, the transferrin receptor has been targeted by transferrin–drug conjugates and peptide-guided nanoparticles to ferry chemotherapeutics across the BBB and into tumor cells (Johnsen et al., 2019; Thomsen et al., 2022). Tumor-specific peptides such as RGD and other homing peptides have also been incorporated into delivery systems to improve glioma targeting in preclinical studies. These efforts, spanning a range of tumor markers, have reinforced the principle that targeted conjugates can achieve concentrated drug action on glioma cells *in vitro* and in animal models.

Despite these promising advances, significant translational challenges remain, including ensuring conjugate stability to prevent premature payload release and unintended systemic toxicity. Optimizing linker chemistry is essential to guarantee stable circulation and selective intracellular drug release. Achieving sufficient target specificity is complicated by glioma heterogeneity and variable antigen expression, which can lead to off-target effects and reduced efficacy. Additionally, poor penetration of large drug conjugates across the BBB limits their distribution within brain tumors, necessitating novel approaches such as BBB-penetrating peptides or focused ultrasound to enhance brain uptake. Lastly, immunogenicity presents another critical obstacle, especially for conjugates containing bacterial toxins or murine-derived antibodies, prompting advances towards humanized antibodies, less immunogenic payloads, and improved formulations to enable repeated dosing and enhance therapeutic effectiveness. Drug conjugates represent a targeted therapeutic approach for glioma therapy with demonstrated preclinical efficacy against key glioma-associated markers. From a clinical perspective, the tumor-specific targeting capability of drug conjugates has significant potential to minimize off-target toxicities, reduce adverse events, and ultimately improve patient tolerability and quality of life. However, successful clinical translation necessitates overcoming substantial challenges regarding conjugate stability, specificity, BBB penetration, and immunogenicity through ongoing technological refinement and strategic innovation.

## 4 Focused ultrasound-assisted drug delivery systems

Focused ultrasound combined with microbubble contrast agents has emerged as a powerful method to transiently and reversibly disrupt the BBB in a targeted manner (Zhu et al., 2024). When ultrasonic waves are focused on a tumor region after systemic microbubble injection, the microbubbles undergo acoustic cavitation, producing mechanical forces generated by microbubble cavitation and oscillation that safely and transiently disrupt tight junctions in the BBB (Shakya et al., 2024). This BBB opening persists only for a short duration before naturally re-sealing, thereby allowing therapeutic agents to penetrate into the brain tumor and surrounding infiltrative zones without permanent damage to the barrier (Mainprize et al., 2019; Englander et al., 2021). Notably, MRI studies in glioma patients have shown that FUS can increase local BBB permeability by 15%–50%, with gadolinium enhancement peaking immediately after sonication and resolving within 20 h (Mainprize et al., 2019). This transient and repeatable BBB modulation is achieved noninvasively or with minimally invasive implanted devices, and with sub-millimeter precision under imaging guidance (Sandor et al., 2014; Durham et al., 2024), making FUS an attractive modality to enhance drug delivery specifically to glioma tissue while sparing healthy brain regions.

Comprehensive preclinical investigations in glioma models have shown that FUS-induced BBB opening can markedly enhance drug delivery and therapeutic effectiveness. In rodent glioma models, FUS has increased intratumoral concentrations of various chemotherapeutics such as doxorubicin, temozolomide, and etoposide by several-fold and improved survival outcomes compared to chemotherapy alone (Carpentier et al., 2024; Seas et al., 2024). For example, Kovacs et al. reported prolonged survival in two glioblastoma mouse models when FUS was used to enhance doxorubicin delivery (Kovacs et al., 2014). Similarly, the use of real-time acoustic feedback control has enabled safe, consistent carboplatin delivery with FUS in a rat glioma model, yielding survival benefits without overt toxicity (McDannold et al., 2019). These encouraging results have led to rapid translation into clinical testing. In the initial stage, Clinical investigations have demonstrated that FUS-mediated disruption of the BBB is both possible and well-tolerated in individuals with glioma (Carpentier et al., 2024). In a landmark first-in-human study, MR-guided FUS was used to open the BBB in glioma patients prior to surgery, resulting in higher drug concentrations in sonicated tumor regions versus unsonicated regions, with no serious adverse events, and the BBB integrity was restored by the next day (Mainprize et al., 2019). Subsequent trials have explored repeated FUS sessions in the therapeutic setting. A recent phase I/II trial in 33 patients with recurrent glioblastoma utilized an implantable ultrasound device (SonoCloud-9) to disrupt the BBB monthly in conjunction with carboplatin chemotherapy (Carpentier et al., 2024). This study achieved BBB opening in 90% of sonications and reported no dose-limiting toxicities, only transient procedure-related grade 1–3 events like headache, fatigue, and minor wound issues. The one-year survival rate in these extensively pre-treated recurrent GBM patients was 58%, with a median overall survival of around 14 months post-resection. Although patient numbers are limited, these clinical findings underscore the potential of FUS to enhance the delivery of chemotherapeutics, macromolecules like antibodies, or nanoparticles to gliomas, warranting further investigation in larger trials.

Despite promising outcomes, several translational barriers must be addressed before clinical integration of FUS-assisted drug delivery in glioma treatment. Ensuring safety is crucial, as excessive ultrasound pressures or microbubble doses can trigger adverse effects like microvascular injury or inflammation (Padilla et al., 2023). Thus, optimal parameters and real-time monitoring techniques are under active investigation. Achieving consistent drug dosage and distribution remains challenging due to variability in administration timing and drug uptake after BBB opening, highlighting a need for standardized protocols to ensure reproducible outcomes (Perolina et al., 2024). Device precision is another concern, patient-specific skull anatomy and acoustic heterogeneity complicate accurate targeting, necessitating advancements like implantable ultrasound emitters and neuronavigation-guided arrays for improved localization (Adams et al., 2021). Appropriate patient selection is also critical, as not all tumors or patient anatomies are equally accessible or responsive to FUS-based therapies, especially in pediatric and midline gliomas (Jovanovich et al., 2023). Finally, developing standardized clinical protocols across diverse settings is essential for reliable therapeutic outcomes and broader clinical adoption (Marques et al., 2024). Addressing these issues through multidisciplinary collaborations will accelerate clinical translation of this promising therapeutic modality for glioma.

## 5 Conclusion

Novel drug delivery systems, including nanoparticle-based carriers, exosome-mediated approaches, drug conjugates, and ultrasound-assisted delivery methods, hold significant promise for overcoming current treatment barriers posed by gliomas, particularly the restrictive nature of the BBB (Table 1). Preclinical studies across these approaches have consistently demonstrated improved drug penetration, enhanced tumor targeting specificity, and significantly decreased systemic toxicity relative to conventional treatments. Despite these advances, substantial translational challenges persist, such as ensuring nanoparticle stability, achieving scalable and reproducible exosome production, optimizing conjugate specificity and immunogenicity profiles, and addressing safety and standardization concerns in ultrasound-assisted delivery protocols. Moving forward, it is crucial for researchers and clinicians to collaboratively address these challenges through multidisciplinary efforts, integrating advances from oncology, neuroscience, bioengineering, and pharmacology. Standardizing protocols, optimizing targeting strategies, improving drug-carrier stability, and conducting rigorous safety evaluations are essential steps to successfully translate these innovative delivery systems from experimental platforms into clinically viable therapies. Moreover, combinational approaches integrating these delivery systems, such as using focused ultrasound to enhance exosome delivery or combining antibody-drug conjugates with BBB modulation, are currently being explored and hold promising potential for significantly advancing glioma therapy. Ultimately, continuous innovation and strategic collaboration in this rapidly evolving field may significantly improve therapy outcomes and quality of life for individuals suffering from glioma.

**TABLE 1:** Mechanisms, advantages, and limitations of each drug delivery approach.

Drug delivery system	Key mechanism	Advantages	Limitations
Exosomes	Natural membrane fusion and endocytosis	Excellent BBB penetration, endogenous targeting	Scalability, cargo heterogeneity, and loading efficiency
Drug Conjugates	Tumor-specific antigen targeting with linker cleavage	High specificity, low systemic toxicity	BBB permeability, immunogenicity, and linker stability
Focused Ultrasound (FUS)	Mechanical disruption of tight junctions via cavitation	Transient BBB opening, site-specific delivery	Technical complexity, need for real-time imaging guidance
